# On the Curability of Malignant Tumors of the Breast by Adequate Operations

**Published:** 1881-01

**Authors:** Samuel W. Gross

**Affiliations:** Philadelphia; Surgeon to and Lecturer on Clinical Surgery in the Jefferson Medical College Hospital and the Philadelphia Hospital


### Selections.

---

### ON THE CURABILITY OF MALIGNANT TUMORS OF THE BREAST BY ADEQUATE OPERATIONS.

By SAMUEL W. GROSS, A.M., M.D.,
Surgeon to and Lecturer on Clinical Surgery in the Jefferson Medical College Hospital and the Philadelphia Hospital.

In my treatise on Tumors of the Mammary Gland, which was issued from the press only a few weeks ago, I have endeavored to lessen, if not overcome, the reproach which has from time immemorial been attached to the removal of malignant diseases of the breast, by calling prominent attention to the modern doctrine of their primarily local nature, and to the possibility of assuring complete recovery by attacking the involved organ with a bold hand.

The malignity, or auto-infectiousness, so to speak, of a mammary neoplasm evinces itself, first, by its extension to the surrounding tissues, through which it continuously enlarges, occasions the development of secondary tumors in its vicinity, and in the case of recurrence or reproduction after removal; second, by the transference of its cells to the associated lymphatic glands, in which they multiply and form growths which not only invade the adjacent structures, but act as additional centers of general infection; and third, by the occurrence of metastatic tumors in remote organs and tissues. These three attributes—namely, the contamination of the neighboring tissues, the implication of the lymphatic glands, and the development of similar growths in the viscera—may be common to a mammary tumor, or one or two alone may be met with; so that there is a scale of malignity for the different neoplasms of this class.

Myxoma and adenoma, of which the one originates in the connective tissue framework of the mamma and the other in the cells of the secreting apparatus, never infect the glands or the viscera; but they are eminently recurrent tumors, and come, therefore, under the classification of the semi-malignant growths of some practical surgeons. Myxoma reproduces itself in thirty-three per cent. and adenoma in fifty per cent. of all cases; so that the removal of the entire breast and its coverings, if they be implicated, and of recurrent growths as fast as they appear, suffices to bring about a cure.

Sarcoma, which is made up of embryonic connective tissue, and is usually described as fibroplastic, fibronucleated, or recurrent fibroid tumor, must be included among the worst of the mammary neoplasms, although it is generally regarded as being of limited malignity, more writers teaching that its tendency is to recur, but losing sight of the fact that it produces metastases. Unlike carcinoma, it does not affect the associated lymphatic glands; but the invasion of the surrounding tissues is shown by local reproduction in sixty-one per cent. of all operations, and post-mortem inspection discloses visceral growths in fifty-seven per cent. of all instances. While recurrence in loco is not so frequent nor so early as in carcinoma, sarcoma is followed by metastatic tumors in seven per cent. more of cases than is that affection. These are startling facts, and I must assume the credit of having been the first to establish them.

In view of the recurring nature of sarcoma and of its marked liability to infect the viscera, the surgeon must interfere early. He should discard partial operations, and make the rule absolute to amputate the entire breast with its investing skin and fat, by a circular incision, dissect off the fascia of the pectoral muscle, and mop the large wound with a strong solution of chloride of zinc, or touch it with the iron at a dull red heat. Recurrent growths must be freely extirpated as rapidly as they appear, since in this way suffering may be alleviated, life be prolonged, visceral contamination be averted, and permanent recovery assured in a certain proportion of cases. In an example of medullary small spindle-celled sarcoma Professor Gross succeeded, after removing fifty-two tumors, by twenty-three distinct operations in four years and a half, in the last of which large portions of the pectoral and intercostal muscles were cut away, in checking reproduction. Nearly eleven years subsequently the woman was entirely well. In similar cases Gay had added nine years to his patient's life at the date of the last report, and Heath and Haward kept their patients alive for thirteen years.

Carcinoma, which is an infiltrating epithelial neoplasm, and is ordinarily known as cancer, is the most malignant of all the tumors of the mamma. Its course is not only more rapid than that of sarcoma, the average duration of life being only thirty-nine months from its first observation to its final termination after operation, against seven years for sarcoma, but it implicates the lymphatic glands in sixty-four per cent. of all cases, recurs in eighty per cent. after extirpation, and occasions metastatic deposits in fifty per cent. of all instances. These properties, along with the discouraging results following incomplete operations, have led some surgeons to refrain from interfering altogether, while others remove the disease with the view merely to avert mental and physical suffering. That both of these practices are erroneous is conclusively shown by the facts, first, that extirpations of the carcinomatous mass add twelve months to the life of the patient; and secondly, that bold measures result in permanent recovery in 10 51 per cent. of all cases.

In the treatise already referred to I use the following language in regard to what I mean by the term cure or permanent recovery: "Metastatic tumors develop in thirty-one months, and death usually ensues, no matter whether the patients have been operated upon or not, in thirty-three months on an average. Local reproduction after removal is witnessed in less than one case out of every hundred after the expiration of three years; so that if the patient survives three years after the last operation without recurrence, or dies of some intercurrent malady under the same circumstances, I assume that she has recovered. Although, of course, each case will have to be dealt with in accordance with its individual merits, the question must be decided by facts based upon the general life of the disease. Of four hundred and eighty-five cases of ordinary scirrhous, medullary, colloid, and atrophying carcinoma, in which the history is complete, fifty-one or 10.51 per cent.—and forty-seven were still living—fulfilled these requirements, the average life after operation having been four years and ten months. Of the cases in which the affection pursued a natural course only 1.5 per cent. survived six years, while of those cured by the knife thirty per cent. were living free from disease after the expiration of six years, four were alive for more than seven years, and the remaining eleven were well for periods which varied between eight and ten years."

The view that carcinoma is in the first instance a local disease, and that it is curable by thorough operations practiced before it has disseminated itself extensively in the adjacent structures or has tainted the general system, is rapidly gaining adherents among some of the best observers of the world, among whom may be mentioned Simon, Moxon, Arnott, Payne, Hutchinson, and Sir William Gull, of England; Nussbaum, of Munich; Fischer, of Breslau; Esmarch, of Kiel; Kocher, of Bonn; and Billroth, of Vienna. Apart from the practical test of the results of surgical interference, the study of the general pathology of the disease shows, first, that it is at the outset a local degeneration of the mamma, and that its tendency is to advance toward the surface before it invades the deeper structures, the lymphatic glands, and the viscera; and secondly, that local infection does not ensue, on an average, before the expiration of thirteen months, the lymphatic glands in fifteen months, the walls of the chest in twenty-two months and the viscera in thirty-one months. Hence if the carcinomatous mamma can be completely gotten rid of before it has contaminated the adjacent structures there is no reason why the remedy should not prove to be final.

When the tumor is of moderate volume, and devoid of superficial and deep attachments and palpable enlargement of the lymphatic glands, the operation which I now invariably practice and earnestly recommend is to remove the entire breast and its coverings by a circular incision, search for any outlaying lobules that may be disseminated throughout the mammary region, dissect off the fascia of the pectoral muscle, and prolong the outer portion of the incision into the axilla with a view to its thorough exploration. Although the glands may have eluded detection previous to surgical interference, careful examination will usually disclose that several are already converted into secondary tumors; and in this event the axillary space must be thoroughly cleaned out, with the object of getting rid of so many independent sources of infection of the adjacent tissues and the associated glands. Ample experience shows, first, that the seats of recurrence, or, rather, further spread of the disease, after operation, are the skin, paramammary fat, remains of the mamma, pectoral fascia, and glands of the axilla; and secondly, that recurrence in the axilla is more frequent by twenty per cent. after removal of the breast alone than when that cavity was freed of its contents simultaneously with the extirpation of the breast. Hence it is that bold measures alone can be depended upon to assure a successful issue.

Even when the skin, pectoral fascia, muscles and glands are implicated, provided the evidences of local dissemination be not too extensive, thorough operations frequently result in permanent recovery. Thus out of forty-eight of the fifty-one cures in which the extent of the operation is noted, in nineteen the entire breast was amputated and the axilla was cleaned out; and in several of these there were nodules in the skin and the upper layer of the great pectoral muscle removed. It is, moreover, comforting to know that the glands may be merely the seat of irritative hyperplasia, since in three cases in which they were permitted to remain the patients were free from recurrence respectively for five years and nine months, six years and one month and ten years and ten months. Glandular involvement is, however, of bad prognostic import, as the chances for permanent recovery are three times greater when the breast alone requires amputation. The same statement is true of extensive infiltration of the pectoral muscles, but these may be cut away with a free hand with some prospect of relief.

As a precaution against local reproduction the wound may be sponged with a strong solution of chloride of zinc or be seared with the hot iron, and the latter agent should always be resorted to when nodules have been cut out of the pectoral or intercostal muscles or the ribs or costal cartilages.

While it is true that adequate operations do not increase the mortality, they are certainly justifiable, as they alone can be relied upon to cure a disease which is so inevitably fatal as carcinoma. In favor of the method it may be said that as the wound is an open one the danger of the retention and putrefaction of discharges and of the evil consequences which follow these accidents is obviated. In my own hands the operation has been attended with the best results, since of ten cases in which I amputated the entire mamma and its coverings, dissected off the pectoral fascia and cleaned out the axilla, all recovered.

